# Alpha Ketoglutarate Exerts In Vitro Anti-Osteosarcoma Effects through Inhibition of Cell Proliferation, Induction of Apoptosis via the JNK and Caspase 9-Dependent Mechanism, and Suppression of TGF-β and VEGF Production and Metastatic Potential of Cells

**DOI:** 10.3390/ijms21249406

**Published:** 2020-12-10

**Authors:** Katarzyna Kaławaj, Adrianna Sławińska-Brych, Magdalena Mizerska-Kowalska, Aleksandra Żurek, Agnieszka Bojarska-Junak, Martyna Kandefer-Szerszeń, Barbara Zdzisińska

**Affiliations:** 1 Department of Virology and Immunology, Maria Curie-Sklodowska University, Akademicka 19, 20-033 Lublin, Poland; katarzyna.kalawaj@op.pl (K.K.); magdalena.mizerska-dudka@poczta.umcs.lublin.pl (M.M.-K.); zurekaleksandra@wp.pl (A.Ż.); kandem@poczta.umcs.lublin.pl (M.K.-S.); 2 Department of Cell Biology, Maria Curie-Sklodowska University, Akademicka 19, 20-033 Lublin, Poland; adrianna.slawinska-brych@poczta.umcs.lublin.pl; 3 Chair and Department of Clinical Immunology, Medical University of Lublin, Chodźki 4a, 20-093 Lublin, Poland; agnieszka.bojarska-junak@umlub.pl

**Keywords:** alpha-ketoglutarate, cell cycle, apoptosis, JNK, cell migration, cell invasion, TGF-β, VEGF

## Abstract

Osteosarcoma (OS) is the most common type of primary bone tumor. Currently, there are limited treatment options for metastatic OS. Alpha-ketoglutarate (AKG), i.e., a multifunctional intermediate of the Krebs cycle, is one of the central metabolic regulators of tumor fate and plays an important role in cancerogenesis and tumor progression. There is growing evidence suggesting that AKG may represent a novel adjuvant therapeutic opportunity in anti-cancer therapy. The present study was intended to check whether supplementation of Saos-2 and HOS osteosarcoma cell lines (harboring a TP53 mutation) with exogenous AKG exerted an anti-cancer effect. The results revealed that AKG inhibited the proliferation of both OS cell lines in a concentration-dependent manner. As evidenced by flow cytometry, AKG blocked cell cycle progression at the G_1_ stage in both cell lines, which was accompanied by a decreased level of cyclin D1 in HOS and increased expression of p21^Waf1/Cip1^ protein in Saos-2 cells (evaluated with the ELISA method). Moreover, AKG induced apoptotic cell death and caspase-3 activation in both OS cell lines (determined by cytometric analysis). Both the immunoblotting and cytometric analysis revealed that the AKG-induced apoptosis proceeded predominantly through activation of an intrinsic caspase 9-dependent apoptotic pathway and an increased Bax/Bcl-2 ratio. The apoptotic process in the AKG-treated cells was mediated via c-Jun N-terminal protein kinase (JNK) activation, as the specific inhibitor of this kinase partially rescued the cells from apoptotic death. In addition, the AKG treatment led to reduced activation of extracellular signal-regulated kinase (ERK1/2) and significant inhibition of cell migration and invasion in vitro concomitantly with decreased production of pro-metastatic transforming growth factor β (TGF-β) and pro-angiogenic vascular endothelial growth factor (VEGF) in both OS cell lines suggesting the anti-metastatic potential of this compound. In conclusion, we showed the anti-osteosarcoma potential of AKG and provided a rationale for a further study of the possible application of AKG in OS therapy.

## 1. Introduction

Osteosarcoma (OS) is a malignant mesenchymal-origin primary tumor of bone. Its histological hallmark is the production of osteoid or immature bone by neoplastic cells [1]. Although bone tumors are relatively rare overall (less than 1% and 3–5% of all newly diagnosed malignant cancers in adults and children, respectively), OS is the most common bone cancer in children and adolescents [2]. OS has a bimodal age incidence distribution worldwide with the first peak in teenagers (at the age of 10–14 in females and 15–19 in males) and the second peak in the elderly [3]. The survival rates may vary depending on various factors (e.g., age, sex, disease stage, localization, country); in children and adolescents, they are similar in most countries, ranging from 55% to 75% [4]. However, the 5-year overall survival rates of OS patients with distant metastasis and/or relapsed OS is low, i.e., approximately 30% in individuals with lung metastasis [5]. The current OS treatment combines surgery with chemotherapy, which was introduced in the 1980s and resulted in significant improvement in OS patients’ survival rates over the 1990s [4]. Most importantly, for the past 20 years, the survival rates in OS patients did not change essentially and no successful targeted therapies of this cancer have been developed so far [6]. Therefore, there is still a need for novel OS treatment strategies. 

Alpha-ketoglutarate (AKG) or 2-oxoglutarate is known mainly as an intermediate of the tricarboxylic acid (TCA) cycle that serves the production of the energetic molecule ATP [7], although it can also be synthesized via other biosynthetic pathways in cells [8]. In the TCA cycle, AKG is formed as a product of isocitrate oxidative decarboxylation catalyzed by NADP-dependent isocitrate dehydrogenase isoforms (IDH1-3) [9]. AKG is characterized as a metabolite with pleiotropic activity due to its metabolic and non-metabolic functions associated with direct involvement in different cellular processes as a biosynthetic substrate, a co-substrate of 2-oxoglutarate-dependent dioxygenases (2-OGDDs), or a signaling molecule [8]. Mounting evidence suggests that AKG is one of the central metabolic regulators of tumor fate and plays an important role in cancerogenesis and tumor development [10]. Most importantly, it is involved in the regulation of hypoxic response and epigenetic modifications, i.e., two main phenomena that drive oncogenic transformation. AKG is a co-substrate for prolyl hydroxylases (PHDs) belonging to the 2-OGDD family, which regulate the stability of the hypoxia-inducible factor (HIF-1), i.e., an important transcription factor in cancer development and progression. Thus, the abundance of AKG may be a determinant of the HIF-1 stabilization/activity through the regulation of PHD activity [11,12]. Moreover, other AKG-dependent enzymes from the OGDD family, namely ten-eleven translocation hydroxylases (TET1-3) and Jumonji C domain-containing lysine demethylases (KDM2-7), are involved in DNA and histone demethylation, respectively, and take part in shaping the cellular epigenetic landscape that is important in cancerogenesis [13]. What is more, mutations in genes encoding TCA cycle enzymes, such as succinate dehydrogenase (SDH), fumarate hydratase (FH), and IDHs, may be present in several cancers, leading to the accumulation of appropriate metabolites, i.e., succinate, fumarate, and D-2-hydroxyglutarate, respectively, inside cells. All these metabolites may act as competitive inhibitors of 2-OGDD enzymes (including PHDs, TETs, and KDMs), and they have been termed “oncometabolites” due to their role in the metabolic reprogramming of cells and progression toward malignancy [14]. It is suggested that an increase in the level of AKG in cells may result in the reverse of 2-OGDD inhibition by oncometabolites and exert an anticancer effect [11,15,16,17,18,19]. 

Recent in vitro and in vivo experiments suggest that an increase in the intracellular level of AKG through different strategies, e.g., exogenous supplementation, alpha-ketoglutarate dehydrogenase (KGD; an enzyme that catalyses the oxidative decarboxylation of AKG to succinyl-Co-A in the TCA cycle) inactivation or even IDH overexpression, may lead to downregulation of HIF-1 or upregulation of epigenetic enzymes and prevent/inhibit tumor progression or show direct anticancer effects [17,19,20,21,22,23,24]. A more recent study has also proposed therapeutic strategies to increase AKG intracellular levels as a mechanism of engagement of latent tumor-suppressive pathways in p53-deficient cancers. It has been shown that AKG is an effector molecule of p53-mediated tumor suppression and its accumulation in p53-deficient tumors can antagonize malignant progression [25].

OS is a cancer with numerous chromosomal abnormalities, gene mutations, and epigenetic defects, e.g., hypermethylation at promoter CpG islands of the key (Rb and p53) tumor suppressor pathways [26]. However, no mutations in the TCA enzymes have been identified so far [27,28] except for one study that has demonstrated IDH2 mutations in OS tissues [29]. Nevertheless, a few studies have shown that IDH1/IDH2 expression inversely correlated with the pathological grade and metastasis in OS [30,31], and the expression of IDH1 was lower in OS than normal bone tissue [24], suggesting that interference in the level of AKG abundance-regulating enzymes may represent a potential target in the OS therapy. Given these reports, our present study was intended to check whether supplementation of OS cells with exogenous AKG exerted an anti-osteosarcoma effect.

## 2. Results

### 2.1. AKG Inhibits Proliferation of OS Cells

**Figure 1:**
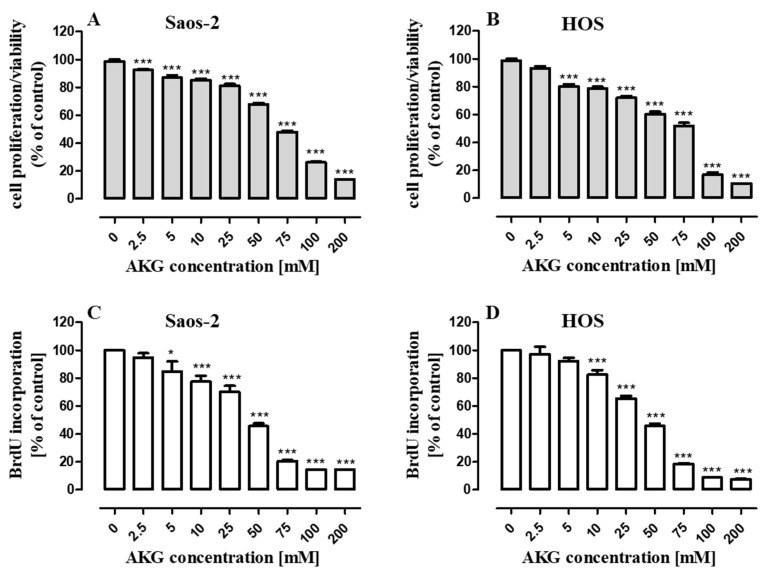
Effect of alpha-ketoglutarate (AKG) on Saos-2 and HOS cell proliferation. The osteosarcoma (OS) cells were treated with increasing concentrations of the compound. Cell proliferation was assessed with the MTT assay after 96 h (**A**,**B**) and the levels of BrdU incorporated into the cells after the 48-h AKG treatment were determined (**C**,**D**). All experiments were repeated independently at least three times, and data (*n* = 24 for each concentration) are expressed as the mean ± SD; * *p* < 0.05 and *** *p* < 0.001 in comparison to the control; one-way ANOVA test.

Since AKG (supplemented as an alpha-ketoglutarate disodium salt dihydrate) was described to exhibit direct antiproliferative activity [22], we evaluated its influence on cell proliferation in two OS cell lines, i.e., Saos-2 (p53-null cell line) and HOS (p53 mutant). The OS cells were cultured in a complete growth medium with AKG, which was used at concentrations ranging from 2.5 to 200 mM established on the basis of research carried out by other authors [20,21,22]. The results showed a concentration-dependent ability of AKG to inhibit proliferation in both cell lines. Incubation of the Saos-2 and HOS cells in a growth medium with increasing concentrations of AKG for 96 h resulted in a similar degree of inhibition of proliferation of both types of OS cells evaluated by the MTT assay (Figure 1A,B). To verify the antiproliferative effect of AKG against OS cells, measurement of DNA synthesis (with the BrdU assay) was additionally performed after 48-h incubation with AKG. As shown in Figure 1C,D, AKG induced a concentration-dependent decrease in OS cell proliferation. The lowest concentrations inducing significant inhibition of the BrdU incorporation into the DNA of dividing cells were 5 mM and 10 mM of AKG for Saos-2 and HOS, respectively. However, the IC_50_ values for both cell lines were very similar and amounted to 35.41 ± 0.17 mM and 35.37 ± 0.19 mM for the Saos-2 and HOS cells, respectively.

### 2.2. AKG Induces Cell Cycle Arrest in the G_1_ Phase in OS Cells through Modulation of the Expression of Cell Cycle-Associated Proteins

**Figure 2:**
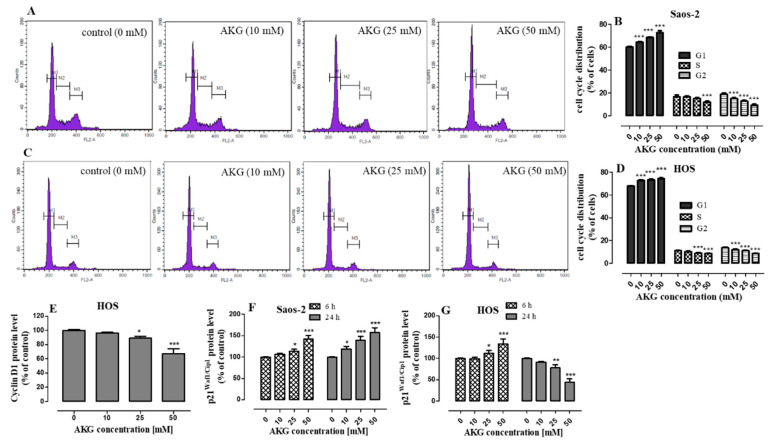
Effect of AKG on cell cycle distribution and expression of cell cycle-associated proteins in Saos-2 and HOS cultures. After the treatment with various concentrations of AKG for 48 h, the cells were stained with propidium iodide and analyzed by flow cytometry. Representative DNA histograms for Saos-2 (**A**) and HOS (**C**) cell lines with statistical analysis of the percentages of cells in the G1, S, and G2 phases in Saos-2 (**B**) and HOS (**D**) cultures. The levels of cyclin D1 in HOS cells (**E**) were measured after 24-h, while p21^Waf1/Cip1^ in the Saos-2 (**F**) and HOS (**G**) cells after 6-h and 24-h incubation without or with AKG (10, 25, and 50 mM) (with the ELISA assay). Data are expressed as means ± SD for at least three independent experiments. (*n* = 3), * *p* < 0.05, ** *p* < 0.01 and *** *p* < 0.001 in comparison to the control; one-way ANOVA test.

To further explore the antiproliferative activity of AKG, the influence of the selected concentrations (10, 25, and 50 mM) of this compound on the distribution of cell cycle phases in both OS cell lines after 48-h incubation was analyzed by flow cytometry. As shown in Figure 2A–D, the AKG treatment at all the concentrations used resulted in accumulation of Saos-2 (Figure 2A,B) and HOS (Figure 2C,D) cells in the G_1_ phase with a concomitant reduction of the cell number in the S and G_2_ phases. Compared to the control, the highest AKG concentration of 50 mM significantly elevated the G_1_-fraction from 60.02 ± 0.92% to 72.81 ± 1.58% in the Saos-2 cell culture and from 67.51 ± 0.29 to 74.37 ± 0.61% in the HOS cell culture.

Since AKG caused the cell cycle arrest in the G_1_ phase, further studies were conducted to investigate the effect of AKG on the expression of proteins responsible for the transition from the G_1_ phase to the S phase of the cell cycle, i.e., cyclin D1 and the cyclin-dependent p21^Waf1/Cip1^ inhibitor. Changes in the expression of these proteins were evaluated by means of immunoassay methods. Only trace amounts of cyclin D1 were detected in the Saos-2 cells (which is in agreement with a previous study [32]), and its expression did not change significantly after the AKG treatment [data not shown]. In contrast, the HOS cells expressed cyclin D1 and its level was downregulated in the AKG-treated cells in a concentration-dependent manner. The 24-h AKG treatment at the concentrations of 25 and 50 mM decreased its expression by approx. 9% and 33%, respectively (Figure 2E). In turn, the expression of the cyclin-dependent p21^Waf1/Cip1^ inhibitor in the AKG-treated Saos-2 cells was significantly upregulated in a concentration- and time-dependent manner. After the 6-h AKG treatment at the concentrations of 10, 25, and 50 mM, the expression of p21 ^Waf1/Cip1^ increased by 7%, 14%, and 42%, respectively. The 24-h treatment with the same concentrations of AKG resulted in a greater increase in the expression of this protein by 19%, 39%, and 57% respectively, in comparison with the control levels (Figure 2F). In the case of the HOS cells, only the 6-h incubation with AKG at the concentrations of 25 and 50 mM induced a statistically significant increase in p21^Waf1/Cip1^ protein expression by 17% and 57%, respectively, compared to the control. In turn, the longer AKG treatment (24 h) resulted in a decrease in the expression of this protein (Figure 2G).

### 2.3. AKG Induces Cell Death in OS Cells through Apoptosis via an Intrinsic Caspase-Dependent Pathway

**Figure 3:**
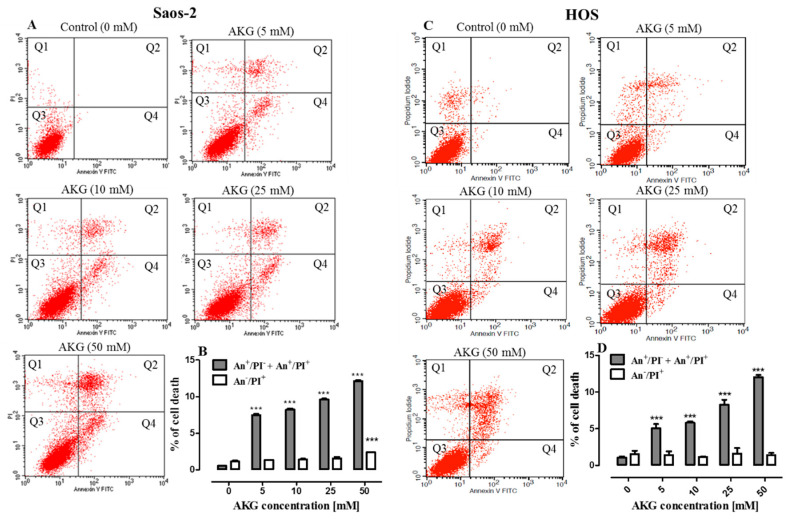
Effect of AKG on apoptosis induction in Saos-2 and HOS cell lines. After the 72-h exposure to the different concentrations of AKG, the cells were stained with annexin (An) V-FITC)/propidium iodide (PI) and examined with flow cytometry. The representative dot plots indicate the percentage of An^−^/PI^+^ necrotic cells (Q1), An^+^/PI^+^ late apoptotic cells (Q2), An^−^/PI^−^ viable cells (Q3), and An^+^/PI^−^ early apoptotic cells (Q4) in AKG-treated Saos-2 (**A**) and HOS (**C**) cell cultures. Histogram representation of the quantitative percentage of total apoptotic cells (early + late apoptosis) and necrotic cells in the control and AKG-treated Saos-2 (**B**) and HOS (**D**) cell cultures. All experiments presented in this figure were repeated independently at least three times, and data (*n* = 12 for each concentration) are expressed as mean ± SD; *** *p* < 0.001 in comparison to the control; one-way ANOVA test.

Since the cell growth inhibition by AKG may have been also a result of the induction of cell death via apoptosis and/or necrosis, the cells were analyzed using Annexin V-FITC/PI double staining and flow cytometry. As shown in Figure 3A–D, after 72-h treatment, AKG was found to induce apoptosis in both Saos-2 and HOS cells, whereas necrosis was only slightly increased when the Saos-2 cells were incubated with 50 mM of AKG (Figure 3B). Significant induction of apoptosis was observed even at 5 mM of AKG. The percentage of Saos-2 cells undergoing apoptosis increased significantly from 0.5 ± 0.01% in the control to 7.5 ± 0.29%, 8.2 ± 0.28, 9.6 ± 0.22%, and 12.1 ± 0.22% after the incubation with 5, 10, 25, or 50 mM of the AKG, respectively (Figure 3B). Similarly, the percentage of HOS cells undergoing apoptosis increased from 1.0 ± 0.16% in the control to 5.0 ± 0,58%, 5.8 ± 0.16%, 8.3 ± 0.30%, and 12.0 ± 0.28% after the incubation with 5, 10, 25, or 50 mM of the compound, respectively (Figure 3D).

**Figure 4:**
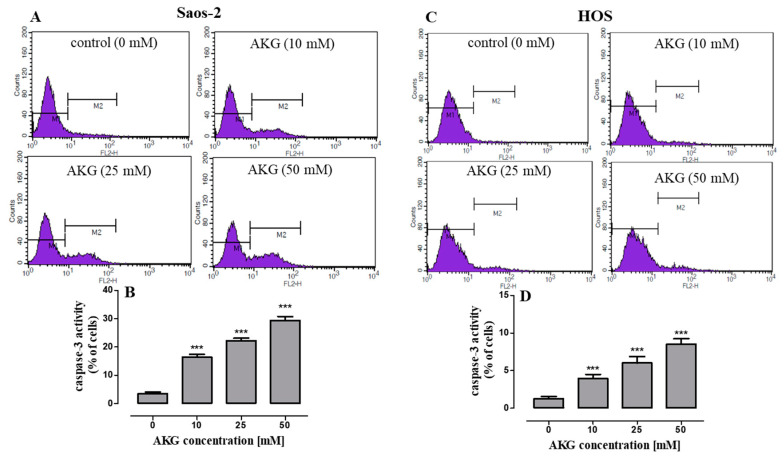
Flow cytometry analysis of active caspase-3 in Saos-2 and HOS cells treated with AKG for 72 h. Representative histograms of Saos-2 (**A**) and HOS (**C**) cell cultures. Symbols M1 and M2 represent peaks for viable (caspase-3 negative cells) and apoptotic cell fractions (caspase-3 positive cells), respectively. Quantification of caspase-3 activity in Saos-2 (**B**) and HOS (**D**) cell cultures. Mean ± SD of 3 measurements in three independent experiments (*n* = 12 for each concentration); statistically significant at *p* < 0.001 *** in comparison to the control; one-way ANOVA test.

Moreover, to identify the mechanism of AKG-induced apoptosis in OS cells, the activation of effector caspase-3 was evaluated by flow cytometry. As shown in Figure 4A–D, in both OS cell lines, the 72-h AKG treatment resulted in a concentration-dependent increase in the number of cells with active caspase 3.

**Figure 5:**
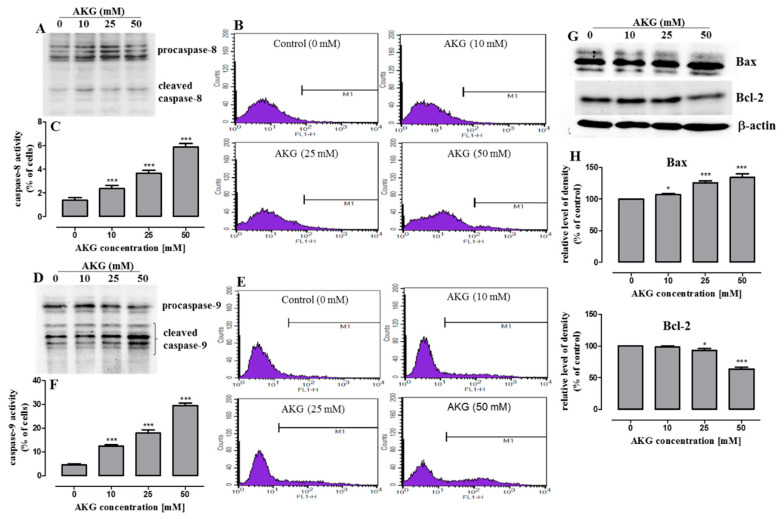
Analysis of the expression of apoptosis-related proteins in Saos-2 cells treated with AKG. After 72-h incubation with AKG, the expressions of procaspases-8 and -9 and cleaved forms of these caspases were examined by Western blotting, and active caspase-8 and -9 were analyzed by flow cytometric analysis. Representative blots from three independent experiments (**A**,**D**). Representative histograms of Saos-2 cell culture (**B**,**E**). Symbol M1 represents peaks for active caspase-8 or -9 positive cells. Quantification of caspase-8 (**C**) and -9 (**F**) activity in Saos-2 cell cultures. Mean ± SD of 3 measurements in three independent experiments (*n* = 12 for each concentration); statistically significant at *** *p* < 0.001 in comparison to the control; one-way ANOVA test. Western blotting of Bax and Bcl-2 expression after 72-h treatment with AKG (**G**) Equal loading was confirmed by immunodetection of *β*-actin. Densitometry analysis of Bax and Bcl-2 bands with ImageLab™ Software (**H**). Data are expressed as means ± SD for at least three independent experiments; (*n* = 3), * *p* < 0.05 and *** *p* < 0.001 in comparison to the control; one-way ANOVA test.

A further study was undertaken to resolve which pathway, receptor- or mitochondria-dependent, was involved in the AKG-induced caspase 3 activation in the OS cells. Since a higher number of cells with activated caspase 3 was observed in the Saos-2 culture, this cell line was chosen to examine the activation of initiator caspase-8 (extrinsic pathway) and caspase-9 (intrinsic pathway) with the use of immunoblotting and flow cytometry methods. As shown in Figure 5A–C, AKG caused a slight increase in the active forms of caspase-8, in comparison with the control, after the 72-h treatment, although only in a small percentage of cells. In contrast, an AKG concentration-dependent decrease in the procaspase-9 levels and an increase in its active form were observed in the Saos-2 cells (Figure 5D), and a large number of cells exhibited the presence of the active form of this caspase (Figure 5E,F). Since these results indicated that the AKG-treatment activated predominantly the intrinsic apoptotic pathway, the expression of pro-apoptotic and anti-apoptotic proteins associated with mitochondrial membrane integrity were further assessed by Western blot analysis at 72 h. As shown in Figure 5G,H, the exposure to AKG triggered a significant increase in the amount of the pro-apoptotic Bax protein and a decrease (although to a lesser extent) in the expression of Bcl-2 (apoptosis inhibitor) in the Saos-2 cells. This suggests that the AKG treatment may result in the predominance of pro-apoptotic signals through an increase in the Bax/Bcl-2 ratio.

### 2.4. AKG Modulates the Phosphorylation of Mitogen-Activated Protein Kinases and Induces Apoptosis in OS Cells through a c-Jun N-Terminal Protein Kinase (JNK)-Dependent Mechanism

**Figure 6:**
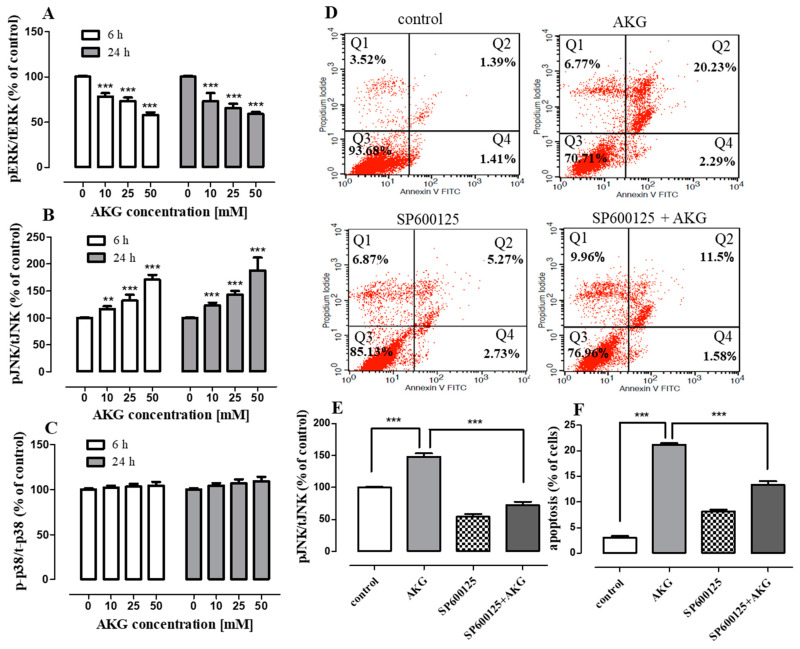
Effect of AKG on phosphorylation of MAP kinases and the influence of the selective JNK inhibitor (SP600125) on AKG-induced apoptosis in Saos-2 cells. The cells were incubated without or with AKG for 6 h and 24 h, and phosphorylated and total ERK1/2, JNK and p38 levels were determined with the ELISA assay. Quantification of the amounts of phosphorylated to total MAP kinases (**A**–**C**). The cells were treated with 50 mM AKG without or with SP600125 (5 μM) and harvested after 72 h of treatment for apoptosis analysis. The representative dot plots indicate the percentage of An^−^/PI^+^ necrotic cells (Q1), An^+^/PI^+^ late apoptotic cells (Q2), An^−^/PI^−^ viable cells (Q3), and An^+^/PI^−^ early apoptotic cells (Q4) in the AKG or/and SP600125-treated Saos-2 cell cultures (**D**). Quantification of the amounts of phosphorylated to total JKN kinase (**E**) and histogram representation of the quantitative percentage of apoptotic (early + late apoptosis) cells (**F**) in the control, SP600125, AKG, and SP600125 + AKG-treated Saos-2 cell cultures. Data are expressed as means ± SD for three independent experiments. ** *p* < 0.01, *** *p* < 0.001 in comparison to the control; one-way ANOVA test.

To explore the involvement of mitogen-activated protein kinases (MAPKs) in AKG-induced OS cell apoptosis, phosphorylation of JNK, extracellular signal-regulated kinase (ERK1/2), and p38 was examined with the quantitative ELISA method. As shown in Figure 6A, the AKG treatment reduced ERK1/2 phosphorylation in a concentration-dependent manner within 6 and 24 h in the Saos-2 cells. In contrast, AKG remarkably augmented the level of phospho-JNK in a concentration-dependent manner, but not phospho-p38 (Figure 6B,C).

JNK is a stress-activated kinase, and a signaling pathway with the participation of this kinase regulates e.g., apoptosis [33]. To clarify whether the AKG-activated JNK signaling pathway was engaged in the apoptotic process, the Saos-2 cells were cultured with AKG in the presence of a specific inhibitor of JNK (SP600125). After 72 h, the percentage of apoptotic cells was measured with FACS, whereas the JNK phosphorylation status was evaluated after 24 h with ELISA. As shown in Figure 6D, the percentage of apoptotic cells declined from 22.52 ± 1.8% after the treatment with 50 mM AKG alone to 13.1 ± 1.2% when the cells were co-treated with AKG and 5 μM of SP600125. It was found that the inhibition of JNK phosphorylation by SP600125 completely inhibited the activation of JNK induced by this compound (Figure 6E) and partially reduced the level of AKG-induced apoptosis in the Saos-2 cells (Figure 6F). These data may therefore support the observation that the AKG-induced apoptosis in the Saos-2 cells was mediated partially through the activation of the JNK signaling pathway.

### 2.5. AKG Inhibits the Migration and Invasiveness of OS Cells and Decreases the Production of VEGF and TGF-β in These Cells

**Figure 7:**
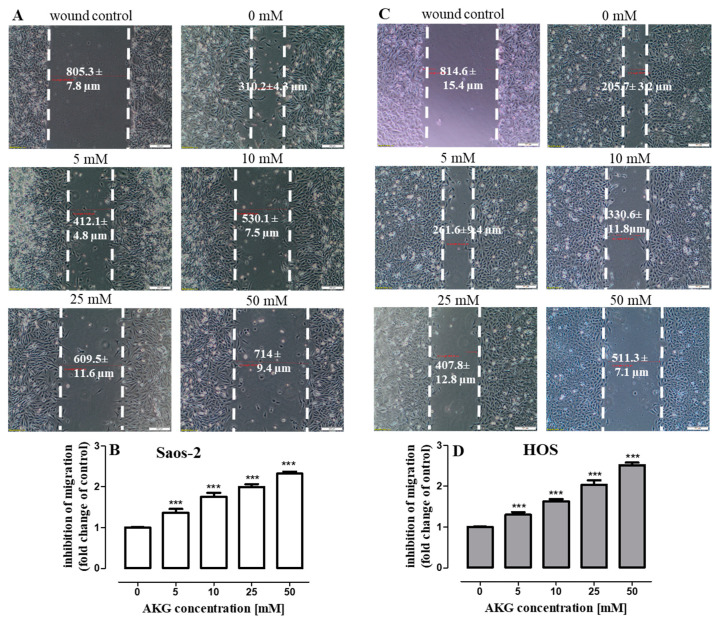
Effect of AKG on migration of Saos-2 and HOS cells in the wound healing assay. Cells were scraped and cultured without or with AKG for 24 h. Saos-2 (**A**) and HOS (**C**) cultures were imaged under a contrast-phase light microscope (magnification ×40) before and after injury. Cell migration was quantified by measuring the gap width of wounds. Quantitative data (**B**,**D**) are presented as a relative fold change in the inhibition of migration in comparison to the control. Data are expressed as means ± SD for three independent experiments. scale bar = 200 µm,*** *p* < 0.001 in comparison to the control; one-way ANOVA test.

**Figure 8:**
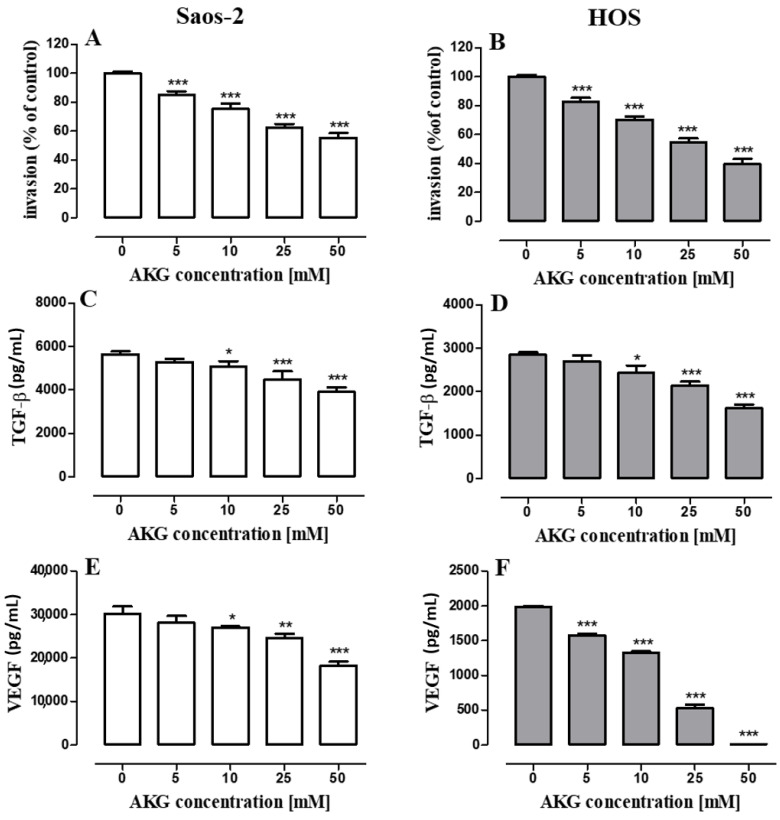
Effect of AKG on cell invasion and production of transforming growth factor β (TGF-**β**) and vascular endothelial growth factor (VEGF) in Saos-2 and HOS cells. Cell invasion was evaluated in a transwell chamber assay with a basement membrane extract (BME)-coated membrane (8 μM) after 24 h. Invaded Saos-2 (**A**) and HOS (**B**) cells were quantified by measuring calcein-AM fluorescence. Following the 72-h AKG treatment, the conditioned media from the Saos-2 and HOS cell cultures were collected and the levels of TGF-β (**C**,**D**) and VEGF (**E**,**F**) were assayed with ELISA. Representative results of three independent experiments are shown. (*n* = 9); statistically significant at *p* < 0.05 *, at *p* < 0.01 ** or at *p* < 0.001 *** in comparison to the control; one-way ANOVA test.

Since OS is classified as a strong tumor metastatic disease, the effect of AKG (5, 10, 25, 50 mM) on the migration (evaluated in a wound-healing assay) and invasion (evaluated in a transwell chamber assay with a basement membrane extract (BME)-coated membrane) of the Saos-2 and HOS cells was assessed. As shown in Figure 7A–D, the AKG treatment suppressed cell migration in both cell lines in a concentration-dependent manner. The inhibition of the migration of Saos-2 and HOS cells after the 24-h AKG treatment at a concentration of 50 mM increased approx. 2.3 and 2.5 times, respectively, in comparison with the migratory potential of control cells. At the same time, the AKG treatment decreased the invasiveness of both cell lines in a concentration-dependent manner (Figure 8A,B). The lowest AKG concentration used, i.e., 5 mM decreased the invasive activity of the Saos-2 and HOS cells by 14 ± 0.87% and 17 ± 2.71%, respectively, in comparison with the control cells. In turn, at the highest AKG concentration, i.e., 50 mM, the invasiveness of the Saos-2 and HOS cells decreased markedly by 43 ± 1.37% and 60.5 ± 3.53%, respectively.

In addition, the production of some cell migration-, invasion- and angiogenesis-associated growth factors such as transforming growth factor β (TGF-β) and vascular endothelial growth factor (VEGF) was examined by ELISA. As shown in Figure 8C,D, both OS cell lines constitutively produced significant amounts of TGF-β, although the Saos-2 cells secreted almost two-fold higher levels of this cytokine than the HOS cells. The level of TGF-β produced by the control Saos-2 and HOS cultures was 5570 ± 27.85 pg/mL and 2851 ± 55.70 pg/mL, respectively. The 72-h treatment of the Saos-2 and HOS cells with AKG suppressed the production of TGF-β in a concentration-dependent manner in both OS cell types (Figure 8C,D). At the highest concentration tested, i.e., 50 mM, AKG decreased the production of TGF-β in the Saos-2 and HOS cells by approx. 29% and 43%, respectively. Similarly, both OS cell lines secreted constitutively significant amounts of VEGF, although also in this case the control Saos-2 cells produced substantially higher quantities of this growth factor than the HOS cells (29 670 ± 35 pg/mL vs. 1985 ± 14 pg/mL, respectively). The 72-h treatment of the Saos-2 and HOS cells with AKG suppressed the production of VEGF in a concentration-dependent manner in both OS cell lines (Figure 8E,F); however, this effect was stronger in the HOS cells. In this cell line, all the AKG concentrations tested, i.e., 5, 10, 25, and 50 mM, inhibited significantly VEGF production by approx. 21%, 33%, 74%, and 94%, respectively (Figure 8F). After the treatment of the Saos-2 cells with AKG at a concentration of 10, 25, and 50 mM, the production of VEGF decreased by approx. 9%, 17%, and 39%, respectively (Figure 8E).

## 3. Discussion

OS is the most common type of primary bone cancer, often associated with a high degree of malignancy, early metastasis, and rapid progression. There are different molecular types of OS; however, the tumor suppressor *TP53* is the most frequently altered gene in OS [6,26]. Although the survival rate in the case of non-metastatic patients is quite high, distant metastases (mainly to lungs) are found in about 20% of OS patients and the prognosis for these patients is still poor due to strong resistance of OS to chemotherapy [34]. Given the high rates of recurrence after tumor resection, resistance to chemotherapy and stagnation in the survival rates of OS patients during the last years, an intensive search for novel agents and alternative strategies to combat this malignancy is suggested [35].

Evidence from recent studies suggests that exogenous supplementation of AKG may exert anti-cancer effects against colorectal carcinoma or breast cancer [19,22]. Moreover, our recent study has revealed that exogenous AKG can inhibit cell proliferation and stimulate differentiation of normal osteoblasts [36]. However, the influence of AKG on osteosarcoma cell lines has not been studied so far. In the present study, to assess the anti-osteosarcoma potential of exogenous AKG, two primary osteosarcoma cell lines harboring *TP53* mutations were used, i.e., Saos-2 (p53-null cell line) and HOS (p53 mutant). The AKG treatment resulted in significantly reduced OS cell proliferation with IC_50_ values of approx. 35 mM for both cell lines (in the BrdU assay). In the study conducted by other authors [22], AKG inhibited DNA synthesis in colon carcinoma cell lines such as Caco-2, HT-29, and LS-150 with IC_50_ values of approx. 55 mM, 64 mM, and 67 mM, respectively. In turn, AKG was able to inhibit slightly the growth of MDA-MB-231 breast cancer cells at a concentration of 1 mM [19], while OS cell proliferation was inhibited by AKG at a concentration of 2.5 and 5 mM (in the Saos-2 and HOS cell lines, respectively). These data suggest a cell type-specific effect of AKG, probably related to different oncogenic pathways in the tested tumor cell lines. It is worth mentioning that exogenous AKG does not easily penetrate into the cell (although this occurs via simple diffusion), thus the intracellular level of this metabolite depends on the extracellular concentration [8] in an in vitro study and probably on the time required for its consumption by the cell. Nevertheless, even cell-permeable AKG derivatives (e.g., dimethyl alpha-ketoglutarate), which are often used to increase the intracellular AKG level, were applied at a concentration of 4 mM [25].

Previous studies conducted by other authors have shown that exogenous AKG (25 and 50 mM) can modulate the expression of cell cycle-related proteins such as cyclin D1 and the inhibitor of cyclin-dependent kinases p21^Waf1/Cip1^ [22]. Cyclin D1 is well known for its role in the response to mitogenic signals and regulation of the G_1_ to S phase transition in the cell cycle. This protein activates cyclin-dependent kinases CDK4 and CDK6, which form active complexes with cyclin D1 and phosphorylate the RB protein, leading to transcriptional activation of genes required for cell division [37]. Cyclin D1 and CDK4 have also been reported to be overexpressed in osteosarcoma and related to its occurrence and development [38,39]. In turn, the p21 protein can function as a regulator of cell cycle progression at the G_1_ checkpoint through binding to cyclin/CDK2 complexes and inhibition of RB phosphorylation or direct interaction with PCNA (proliferating cell nuclear antigen), which both trigger inhibition of DNA replication [40]. P21 has been shown to be involved in both p53-dependent and p53-independent control of cell proliferation, differentiation, and cell death [40]. Recently, p21 has been found to be significantly downregulated in osteosarcoma tissue, compared to their matched adjacent non-tumor tissues [41], and upregulation of this protein has been shown to inhibit proliferation of OS cells [42,43]. In our study, AKG (25 and 50 mM) was able to decrease cyclin D1 expression in the HOS cells but not in the Saos-2 cells, which do not express cyclin D1 [32]. On the other hand, AKG remarkably upregulated p21 only in the p53 null OS cells, but not in HOS cells harboring a *TP53* mutation. The results of our study suggest that AKG can disturb cell cycle progression through different mechanisms depending on the distinct genetic characteristics of OS cells.

A successful OS therapy requires, among others, effective agents that promote apoptotic cell death [44]. Drug-induced apoptosis can often occur through extrinsic or intrinsic pathways involving the activation of initiator caspase-8 and -9, respectively. The initiation of the mitochondrial pathway is under the control of the Bcl-2 family members, such as pro-apoptotic Bax and anti-apoptotic Bcl-2, and the Bax/Bcl-2 ratio is a critical determinant of the cell’s apoptotic threshold. Bax insertion into the outer mitochondrial membrane leads to its permeabilization and release of various apoptotic proteins, which trigger the activation of caspase 9/3 signaling cascade [45]. In the present study, the AKG treatment induced apoptosis in both OS cell lines through activation of caspase 3. The further study revealed that the AKG-treatment of the Saos-2 cells resulted mainly in the upregulation of Bax, suggesting that changes in the ratio of Bax/Bcl-2 proteins could contribute to the subsequent activation of caspases-9 and -3. Surprisingly, a similar mechanism associated with the induction of apoptotic death was mediated by upregulation of IDH1 in osteosarcoma cell lines [24].

Mitogen-activated protein kinases (MAPKs), such as extracellular signal-regulated kinases (ERK1/2), c-Jun N-terminal protein kinases (JNKs), and p38, have been identified as key proteins of the signaling pathways that transmit mitogenic signals into the nucleus in response to various extracellular stimuli [46]. The ERK pathway is usually identified as a key mediator of cell proliferation and survival [47]. In turn, JNK may play a dual role in cancer cell survival, but preferentially exerts a pro-apoptotic effect [33,46,48]. Mounting evidence indicates that activation of JNK kinase in response to various anti-cancer agents may contribute to OS cell death [49,50]. In our study, AKG induced phosphorylation of JNK in the Saos-2 cells. Moreover, the pretreatment of the OS cells with the JNK inhibitor abolished the AKG-induced increase in the phosphorylation of this kinase and partially inhibited the AKG treatment-induced apoptosis. These data suggest an essential role of the JNK signaling pathway in AKG-induced apoptotic death of OS cells. It is well known that activated JNK promotes an intrinsic apoptotic pathway and cytochrome c release from the mitochondrion via multiple mechanisms, including Bax and Bcl-2 regulation [33]. More importantly, it phosphorylates cytoplasmic Bax-anchor proteins, which triggers dissociation of Bax from the complexes, its translocation to mitochondria, and induction of outer mitochondrial membrane permeabilization [51]. Moreover, JNK may increase the expression of Bax through transcriptional activation of c-Jun [52]. Furthermore, it may induce apoptosis through direct Bcl-2 phosphorylation and inhibition of its anti-apoptotic activity [33]. Since the AKG treatment of the Saos-2 cells resulted in both JNK activation and reduction of the Bax/Bcl-2 ratio, we can suppose that the caspase-9 and caspase-3 activation observed was linked with the mechanisms above mentioned.

The present study also revealed that AKG decreased ERK1/2 phosphorylation in the Saos-2 cells. The ERK pathway mediates several upstream signals from growth factors (e.g., vascular endothelial growth factor, VEGF) or proinflammatory stimulants, and regulates cell proliferation, migration, and metastasis in most cancers, including osteosarcoma [53]. It has been shown that overexpression and abnormal activation of the ERK signaling pathway are implicated in the pathogenesis of OS; therefore, this pathway is an attractive molecular target in OS [53,54,55,56]. Many studies have shown that suppression of this pathway by anticancer agents results in increased apoptosis and decreased metastasis in OS [53,57,58]. Therefore, besides the JNK pathway, inhibition of ERK1/2 activation may also be implicated in the AKG-mediated inhibition of cell cycle progression and programmed OS cell death observed in our study. However, this issue needs further investigations, to confirm these suggestions. On the other hand, the decrease in the ERK1/2 activation may be also implicated in the anti-migratory and anti-invasive effects of AKG observed in the OS cells.

Earlier studies have shown that exogenous AKG has the ability to reduce the level of the HIF-1α subunit, resulting in downregulation of HIF-1 downstream targets, including the production of VEGF, and inhibition of angiogenesis [20,21]. Moreover, a recent study has shown that exogenous supplementation of AKG prevented tumor growth and metastasis of breast cancer cells through stabilization of PHD2 and decreasing HIF-1α [19]. Furthermore, other strategies associated with intracellular AKG accumulation resulted in anti-metastatic effects [17,24]. In the present study, the AKG supplementation also markedly inhibited cell motility and invasion of both OS cell lines in a concentration-dependent manner, which confirms the anti-metastatic potential of this compound. Moreover, AKG was able to decrease the production of TGF-β and VEGF by the OS cells, i.e., growth factors that are implicated in osteosarcoma progression and metastasis.

Although TGF-β acts in most cancers both as a tumor suppressor in premalignant stages and a tumor promoter in advanced stages of the disease, in OS it exerts only pro-tumoral effects through the promotion of metastasis [59]. It has been shown that the level of TGF-β in sera of OS patients is higher compared to those of healthy donors, which is correlated with a high grade of disease and associated with chemoresistance and presence of metastases in lungs and other sites [60]. Moreover, in vitro studies have revealed that TGF-β is implicated in the EMT-like phenomenon, stimulates proliferation of OS cells, and exerts pro-angiogenic properties in OS [60,61]. In addition, the secretion of TGF-β by OS cells or stromal cells can regulate the phenotype and function of the microenvironment in order to stimulate switching its function to pro-tumoral [59]. Since TGF-β plays a pro-tumoral role in OS, the downregulation of TGF-β by AKG seems to be an important feature of this compound in terms of anti-cancer activity.

In the present study, AKG was also able to inhibit the VEGF production in both OS cell lines, which is in agreement with the results of previous studies conducted by Matsumoto et al. [20,21] in the Hep3B hepatocellular carcinoma cell line and LCC (Lewis lung carcinoma) cell line. In their study, AKG decreased VEGF production at a concentration of 7.5 mM and 5 mM, respectively. Similarly, in our study, the lowest concentrations inducing significant inhibition of VEGF production were 5 mM and 10 mM of AKG for HOS and Saos-2 cells, respectively. VEGF is a key potent tumor-derived pro-angiogenic factor influencing both the tumor microenvironment and cancer cells. It acts in a paracrine manner on endothelial cells which leads to the promotion of angiogenesis [62]. Moreover, VEGF can act in an autocrine manner on several cancer cells, including aggressive osteosarcoma phenotypes, which leads to activation of various signaling pathways in these cells (e.g., PI3K/Akt and MEK/ERK), ultimately supporting tumor growth [63]. VEGF plays an important role in the pathogenesis of OS [64]. VEGF serum levels in OS patients are elevated and associated with poor prognosis [65]. Moreover, overexpression of VEGF is a predictor of pulmonary OS metastasis [66,67]. Recently, it has been shown that silencing of VEGF in Saos-2 cells inhibited cell proliferation and promoted apoptosis in vitro [68]. Therefore, the inhibition of VEGF production in osteosarcoma cells by AKG treatment may have therapeutic value.

As mentioned earlier, we used cell lines with p53 function deficiency in our study; however, the *TP53* gene mutation does not occur in all OS cases. Early studies reported that the rate of the *TP53* gene mutation in OS is around 20% [69]; however, recent studies have shown that more than 90% of osteosarcomas have either missense mutations in this gene or structural variation in p53 [70]. Nevertheless, some percentages of osteosarcomas have functional p53, which raises a question of whether AKG would act in these cases in a similar way as in cells harboring the *TP53* mutation. A recent study has shown that AKG is an effector molecule of p53-mediated tumor suppression, and its accumulation in p53-deficient tumors can partially recapitulate the p53 action linked with the remodeling of cancer cell metabolism through epigenetic modifications and alterations of gene expression [25]. It has also been shown that exogenous AKG can switch metabolism from glycolytic to oxidative, which can prevent the growth and metastasis of breast cancer cells [19]. Based on the analysis of cell lines in which AKG exhibited anti-cancer activity [19,20,21,22], we identified that all these cell lines, except the LS-180 colon cancer cell line, have *TP53* mutations; nevertheless, AKG also inhibited the proliferation of LS-180 cells with the wild type of p53. Therefore, we can speculate that AKG would similarly affect OS lines with functional p53, and that more than one mechanism of AKG activity may operate in cancer cells, depending on the distinct genetic/epigenetic characteristics of these cells. However, further studies of the AKG influence on OS cell lines with functional p53 and their metabolism are needed to confirm this hypothesis.

## 4. Materials and Methods

### 4.1. Cell Culture and AKG

Human osteosarcoma cell lines Saos-2 (HTB-85^TM^) and HOS (CRL-1543^TM^) were purchased from the American Type Culture Collection (ATCC, Manassas, VA, USA). The Saos-2 cells were maintained in McCoy’s 5A Modified Medium (Sigma-Aldrich Chemicals, St. Louis, MO, USA) supplemented with an antibiotic/antimycotic solution (a/a; Sigma-Aldrich) and 10% fetal bovine serum (FBS; Sigma-Aldrich) in a 5% CO_2_ humidified atmosphere at a temperature of 37 °C. The HOS cells were grown in Eagle’s Minimum Essential Medium (Sigma-Aldrich) supplemented with 10% FBS and a/a. The cells were maintained in a humidified incubator with 5% CO_2_ in air at 37 °C.

Alpha-ketoglutarate disodium salt dihydrate (Na_2_AKG × 2H_2_O; (Sigma-Aldrich) was used in the experiments. Before each experiment, the stock solution of AKG (1 M) was prepared by dissolving the compound in the culture medium. The stock solution was filtered through a sterile syringe filter Millex-GV (Merck Millipore Corporation, Burlington, MA, USA) and diluted in an appropriate culture medium to obtain the required concentrations.

### 4.2. Cell Proliferation Assays

The influence of AKG on the proliferation of OS cells was estimated with the MTT (3-(4,5-dimethylthiazol-2-yl)-2,5-diphenyltetrazolium bromide solution) assay as described previously [71]. Briefly, 4 × 10^3^ cells/well were seeded into 96-well plates in growth medium. After 24 h, the culture medium was removed and the cells were exposed to the dilutions of AKG (2.5–200 mM) prepared in the growth medium with 10% FBS. Following 96-h exposure, the cells were incubated for 3 h with an MTT (Sigma-Aldrich) solution (5 mg⁄mL) and then formazan crystals were solubilized overnight by adding SDS buffer (10% SDS in 0.01 N HCl). The absorbance was determined at a wavelength of 570 nm using an EL800 Microplate Reader (BioTek Instruments, Winooski, VT, USA).

The anti-proliferative activity of AKG was also assessed with the BrdU assay, in which DNA synthesis in proliferating cells was determined after 48 h by measuring bromodeoxyuridine incorporation using a commercial Cell Proliferation ELISA BrdU kit (Roche Molecular Biochemicals, Mannheim, Germany) according to the manufacturer’s instructions.

The absorbance of the control wells was taken as 100% and the results were expressed as a percentage of the control. IC_50_ values were defined as drug concentrations necessary to inhibit 50% of growth, compared to untreated control cells. IC_50_ was obtained using the non-linear regression program GraphPad Prism v5 software (GraphPad Software Inc., San Diego, CA, USA).

### 4.3. Cell Cycle Analysis

The Saos-2 or HOS cells were seeded into 6-well plates in medium containing 10% FBS without or with AKG (10, 25, or 50 mM). After 48-h treatment, the cell cycle analysis consisting of determination of DNA contents on the basis of PI staining was performed using a flow cytometer (BD FACSCalibur, BD Biosciences, San Jose, CA, USA) and the Cell Quest Pro Version 6.0. for the Macintosh operating system as described previously [72]. Briefly, the cells were washed with PBS and fixed overnight with 80% ethanol at −20 °C. Next, the cells were washed with PBS and stained with PI using PI/RNase Staining Buffer (BD Biosciences, BD Pharmingen™, San Jose, CA, USA) for 30 min in darkness at RT. Next, the stained cells were analyzed using FACS Calibur. In total, 10,000 events were measured per sample. The data were analyzed to determine the percentage of cells at each phase of the cell cycle (G1, S, and G2/M).

### 4.4. Flow Cytometry

The quantitative analysis of AKG-induced cell death was performed using an Annexin V-fluorescein isothiocyanate (FITC)/propidium iodide (PI) apoptosis kit (BD Biosciences, BD Pharmingen™, San Jose, CA, USA) and the flow cytometry method as described previously [73]. Briefly, the Saos-2 or HOS cells were seeded into 6-well plates at a density of 7 × 10^5^ cells/well. The next day, the growth medium was replaced with a fresh one containing 2% of FBS supplemented with AKG (5, 10, 25, or 50 mM). In some experiments, the Saos-2 cells were exposed to AKG (50 mM), SP600125 (a selective inhibitor of JNK, 5 μM, Sigma-Aldrich), or a combination of these two compounds. After 72-h incubation, the samples were harvested, washed with PBS, and resuspended in 1 × binding buffer. The cells (1 × 10^5^) were then stained with 5 mM of FITC-Annexin V and 5 mM of PI. After 15-min incubation in the dark at room temperature, the cells were immediately analyzed using a flow cytometer (BD FACSCalibur) with CellQuest Pro Version 6.0 software. All experiments were performed in triplicate and yielded similar results.

The fluorescence-activated cell sorting (FACS) technique was also employed to determine the active form of caspase-3, caspase-9, and caspase-8 in the AKG-treated or untreated OS cells. After 72-h exposure to AKG, a phycoerythrin (PE) Active Caspase-3 Apoptosis Kit (BD Biosciences, San Jose, CA, USA)) and a fluorescein CaspaTag Caspase 9 In Situ Assay kit (Sigma-Aldrich) or a CaspaTag Caspase 8 In Situ Assay kit (Sigma-Aldrich) were used according to the manufacturer’s instructions.

### 4.5. Immunoblotting Analysis

The immunoblotting analysis was carried out as described previously [72]. Briefly, the Saos-2 cells (3 × 10^5^ cells/mL) were seeded into 6-well plates and, after attachment, cultured in medium with 2% FBS without AKG or with AKG for 72 h. The cells were harvested and lysed for 40 min on ice in RIPA buffer (Sigma-Aldrich) supplemented with protease and phosphatase inhibitor cocktail (Sigma-Aldrich) and centrifuged (10,000× *g* for 10 min at 4 °C). The total protein concentrations were determined using a BCA protein assay kit (Pierce^®^ BCA Protein Assay Kit, Thermo Scientific, Rockford, IL, USA). For Western blot analysis, supernatants of RIPA cell lysates were solubilized in 4 x Laemmli sample buffer (Bio-rad Laoratories Inc., Hercules, CA, USA) and denaturized (for 5 min at 100 °C). Equal amounts of the protein extracts (40 micrograms) were electrophoresed on SDS-PAGE (Bio-Rad Laoratories Inc., Hercules, CA, USA, Mini-Protean^®^ Tetra Cell) and transferred onto a PVDF membrane (Merck Millipore Corporation, Burlington, MA, USA). After blocking with 5% non-fat dry milk/TBS/0.1% Tween (Sigma-Aldrich) for 1h at RT and washing, the membranes were incubated overnight at 4 °C with the following primary antibodies: anti-caspase-8 and anti-caspase-9 (1:400) as well as anti-Bcl-2 and anti-Bax (1:1000) (all antibodies from Santa Cruz Biotechnology Inc. CA, USA). Next, primary antibodies were detected by horseradish peroxidase (HRP)-conjugated rat, mouse, or goat secondary antibodies (1:2000, Santa Cruz Biotechnology Inc. CA, USA), and protein-antibody complexes were visualized with the ECL^TM^ Western Blotting Analysis System (Amersham^TM^ GE Healthcare, Buckinghamshire, UK) and Molecular Imager^®^ ChemiDoc^TM^ XRS^+^ (Bio-rad Laoratories Inc., Hercules, CA, USA) equipped with ImageLab^TM^ Version 3.0 Software. The blots were reprobed with antibodies against β-actin (1:500, Santa Cruz Biotechnology Inc., CA, USA) used as a load control. Additionally, protein molecular markers (Precision Plus Protein^TM^ Dual Color Standards, Bio-rad Laoratories Inc., Hercules, CA, USA) were loaded onto electrophoretic gels to control the molecular weight of protein bands. Densitometric measurement of chemiluminescent signals was performed using software ImageLab^TM^ Version 3.0. The optical density of the bands was normalized to β-actin levels.

### 4.6. ELISA Assays

The Saos-2 or HOS cells (3.3 × 10^5^ cells/mL) were incubated for 72 h without or with AKG (5, 10, 25, 50 mM) in 24-well plates containing appropriate culture medium with 2% FBS at 37 °C in an atmosphere of 5% CO_2_. Enzyme-linked immunosorbent assay (ELISA) kits were used to measure the levels of TGF-β (DRG International Inc., Springfield, NJ, USA) and VEGF (Diaclone Besançon, France) in the culture media according to the manufacturer’s instructions.

### 4.7. PathScan ELISA Assays

The quantification of the intracellular levels of total and phosphorylated JNK, ERK1/2, p38, and AKT kinases and the contents of cyclin D1 and p21 proteins in the treated cells was carried out using the PathScan^®^ ELISA kits: Total SAPK/JNK Sandwich ELISA Kit, Phospho-SAPK/JNK (Thr183/Tyr185) Sandwich ELISA Kit, Total p44/42 MAPK (Erk1/2) Sandwich ELISA Kit, Phospho-p44/42 MAPK (Thr202/Tyr204) Sandwich ELISA Kit, Phospho-p38 MAPK (Thr180/Tyr182) Sandwich ELISA Kit, Total Cyclin D1 Sandwich ELISA Kit, Total p21^Waf1/Cip1^ Sandwich ELISA Kit (Cell Signaling Technology Danvers, MA, USA), and p38 MAPK alpha ELISA Kit (Abcam, Cambridge, UK) according to the manufacturer’s instructions as described previously [36]. Briefly, the Saos-2 cells (1 × 10^6^ cells/mL) were incubated in culture medium (2% FBS) without or with the selected concentrations of AKG (10, 25, and 50 mM) in 10-cm diameter plastic plates. In some experiments, the cells were pre-treated with SP600125 (a selective inhibitor of JNK1/2, Sigma-Aldrich) at a concentration of 5 μM for 1 h. After 6, 24, or 48 h of incubation, the media were removed and the cells were rinsed with ice-cold PBS (Sigma-Aldrich). Then, the cells were lysed in lysis buffer (included in the kits) supplemented with PMSF (Sigma-Aldrich) and protease and phosphatase inhibitor cocktail (Sigma-Aldrich) according to the manufacturer’s protocol. Total cell lysates were centrifuged at 14,000× *g* rpm, 5 min at 4 °C, and kept at −80 °C until analysis. Before the assay, the total protein concentrations in the cell lysates were determined with a Pierce BCA Protein Assay Kit (Thermo Fisher Scientific, Waltham, MA, USA), and samples containing equal amounts of total proteins per 100 µL of the sample diluent were subjected to ELISA. The optical density was measured using an E-max Microplate Reader (Molecular Devices Corporation, Menlo Park, CA, USA).

### 4.8. Cell Migration Assay

The wound-healing assay was used to evaluate the influence of AKG on the migration of osteosarcoma cells in vitro. The Saos-2 (3 × 10^5^ cells/mL) and HOS (2.5 × 10^5^ cells/mL) cells were seeded into 3-cm diameter plastic plates and cultured until confluence. Afterwards, wounds were made in the monolayers with a sterile 100-μL pipette tip. The cells were washed twice with PBS to remove cell debris, and fresh growth media without or with AKG (5, 10, 25, and 50 mM) were added. The gap width of the wound in one dish (wound control) was measured and recorded immediately after wounding. After 24-h incubation, the gap width of the wounds in the other plates was measured and contrast-phase images were taken using an inverted microscope Olympus CKX41 (Olympus Optical Co, LTD, Tokyo, Japan) and analyzed with Image Processing (CellSans) software. The inhibition of migration (fold change of the control) was calculated as the quotient of the mean width of the gap in the AKG-treated culture and the mean width of the gap in the control culture.

### 4.9. Cell Invasion Assay

The OS cell invasion was estimated with the CultureCoat^®^ 24 Well Low BME Cell Invasion Assay (Trevigen, Inc., Gaithersburg, MD, USA) according to the manufacturer’s instructions. The Saos-2 or HOS cells (both 1.0 × 10^6^ cells/mL) suspended in medium with 1% of FBS and supplemented with AKG (5, 10, 25, or 50 mM) or without AKG (control) were seeded into BME-coated inserts (8 µm) in the wells of a 24-well plate. Medium with 10% FBS (as a chemoattractant) was added into the bottom chamber. After 24 h of incubation, the number of OS cells invading the bottom surface of the insert membrane was quantified based on the amount of free Calcein generated from Calcein AM by migrating cells, as indicated in the manufacturer’s instructions. The Calcein fluorescence level was measured by means of a Perkin Elmer Victor™ plate reader (PerkinElmer, Waltham, MA, USA)

### 4.10. Statistical Analysis

Each experiment was repeated at least three times. Statistical analyses were performed using GraphPAD Prism 5 (GraphPAD Software Inc., San Diego, CA, USA). The data were analyzed by one-way ANOVA followed by Dunnett’s or Tukey’s multiple comparison tests. Values were expressed as means ± SD, and *p* values < 0.05 were considered significant.

## 5. Conclusions

In conclusion, our data demonstrated the anti-osteosarcoma effects of AKG supplementation in an in vitro study. AKG was able to modulate the expression of cell cycle-associated proteins (cyclin D1, p21^Waf1/Cip1^) and arrest cell cycle progression at the G_1_ phase, which resulted in inhibition of OS cell proliferation. Moreover, the AKG-induced activation of the JNK pathway, augmentation of the Bax/Bcl-2 ratio, and activation of caspase-9 and -3 led to the induction of apoptotic cell death in the OS cells. The inhibition of the ERK pathway by AKG may also be involved both in the pro-apoptotic effect of AKG and in the anti-metastatic potential of AKG linked with inhibition of OS cell motility and invasion by this compound. The anti-osteosarcoma potential of AKG was also attributed to its inhibitory influence on the production and release of cytokines such as pro-metastatic TGF-β and pro-angiogenic VEGF. These results may thus provide a rationale for further in vivo study of the possible application of AKG in osteosarcoma therapy.
